# A novel selective medium for the isolation of *Burkholderia mallei* from equine specimens

**DOI:** 10.1186/s12917-019-1874-0

**Published:** 2019-05-07

**Authors:** Yuta Kinoshita, Ashley K. Cloutier, David A. Rozak, Md. S. R. Khan, Hidekazu Niwa, Eri Uchida-Fujii, Yoshinari Katayama, Apichai Tuanyok

**Affiliations:** 1 Microbiology Division, Equine Research Institute, Japan Racing Association, 1400-4 Shiba, Shimotsuke, Tochigi, 329-0412 Japan; 2 Department of Infectious Diseases and Immunology, College of Veterinary Medicine, University of Florida, Gainesville, FL 32608 USA; 3 Unified Culture Collection, Diagnostic Systems Division, U.S. Army Medical Research Institute of Infectious Diseases, Fort Detrick, Frederick, MD 21702-5011 USA

**Keywords:** Bacterial isolation, *Burkholderia mallei*, Equine, Glanders, Horse, Selective medium

## Abstract

**Background:**

*Burkholderia mallei* is a Gram-negative bacterium that causes glanders, a zoonotic disease, especially in equine populations (e.g. horses, donkeys, and mules). *B. mallei* usually grows slowly on most culture media, and this property makes it difficult to isolate from clinical specimens. One of the problems is that *B. mallei* is easily overgrown by other bacteria, especially in animal specimens collected from non-sterile sites. The aim of this study was to develop a new selective agar for the laboratory diagnosis of glanders. We formulated a new agar, named BM agar, to enrich *B. mallei* growth, but inhibit the growth of other bacteria and fungi based on their antimicrobial profiles. We compared the growth of *B. mallei* on BM with Xie’s and PC agars, the two previously described selective agars for *B. mallei.*

**Results:**

BM agar could sufficiently grow almost all of the tested *B. mallei* strains within 72 h: only one out of the 38 strains grew scantly after 72 h of incubation. BM agar was further tested with other *Burkholderia* species and various bacterial species commonly found in the nasal cavities and on the skin of horses. We have found that other *Burkholderia* species including *B. pseudomallei* and *B. thailandensis* can grow on BM agar, but non-*Burkholderia* species cannot. Furthermore, the specificities of the three selective agars were tested with or without spiking *B. mallei* culture into clinical specimens of non-sterile sites collected from healthy horses. The results showed that BM agar inhibited growths of fungi and other bacterial species better than PC and Xie’s agars. We have also found that growth of *B. mallei* on BM agar was equivalent to that on 5% horse blood agar and was significantly greater than those on the other two agars (*P* < 0.05).

**Conclusions:**

We believe that BM agar can be used to efficiently isolate *B. mallei* from mixed samples such as those typically collected from horses and other contaminated environments.

**Electronic supplementary material:**

The online version of this article (10.1186/s12917-019-1874-0) contains supplementary material, which is available to authorized users.

## Background

*Burkholderia mallei* is a Gram-negative bacterium that causes glanders, a zoonotic disease. Glanders is a significant contagious disease, particularly in equine populations (e.g. horses, donkeys, and mules), and other animals (e.g. camels, bears, wolves, and dogs), including humans, are known to be susceptible to *B. mallei* infection [1]. The acute form of glanders is typically observed in donkeys and mules with high fever, respiratory symptoms, and death occurring within a few days. In horses, chronic cases usually develop and horses may endure for several years. Chronic glanders increases the risk of contagion because of the prolonged shedding of *B. mallei* [1]. International horse transport is becoming more common and, therefore, quarantine testing (e.g. serological test, molecular diagnosis, and bacterial isolation) before and after the transport is very important to prevent the spread of glanders.

Bacterial isolation is a gold standard method for diagnosing glanders [2]. *B. mallei* can be grown on most enriched media, including sheep blood agar and trypticase soy agar, but grows very slowly, requiring up to 72 h of incubation [3]. This property makes it difficult to isolate *B. mallei* from clinical and environmental specimens because *B. mallei* is easily overgrown by other bacteria, especially in animal specimens collected from non-sterile sites [4, 5]. Therefore, it is recommended to attempt the bacterial isolation from unopend and uncontaminated lesions. However, aseptically collecting specimens is usually difficult because many bacteria and fungi exist as normal flora in respiratory tracts and on skin where the common infections occur. [6, 7]. Additionally, *B. mallei* is often overgrown by other bacteria even in fresh samples obtained under sterile conditions [8].

A previous report compared four selective media and recommended *Pseudomonas cepacia* agar (PC agar) for the isolation of *B. mallei* [9]. However, the report also identified important limitations of PC agar, which could lead to difficulties in diagnosing glanders. Four out of 20 *B. mallei* strains could not grow at all on PC agar and the sizes of 16 colonies, which grew on the agar were quite small (< 1 mm). Similarly, another study has reported that three selective agars, which are basically used for isolation of *B. pseudomallei*, could not grow 20–60% of the assayed *B. mallei* strains [10]. Although another semi-selective agar for *B. mallei* was reported by Xie et al. [3, 11], the effectiveness of this agar has not been compared with those of other media.

In this study, we developed a new selective medium (*Burkholderia mallei* agar: BM agar) for laboratory diagnosis of glanders and compared its bacterial selectivity and ability to grow *B. mallei* with previously reported media by testing with bacterial cultures and horses’ nasal and skin specimens.

## Results

### Bacterial growth on BM agar with pure cultures

**Fig. 1:**
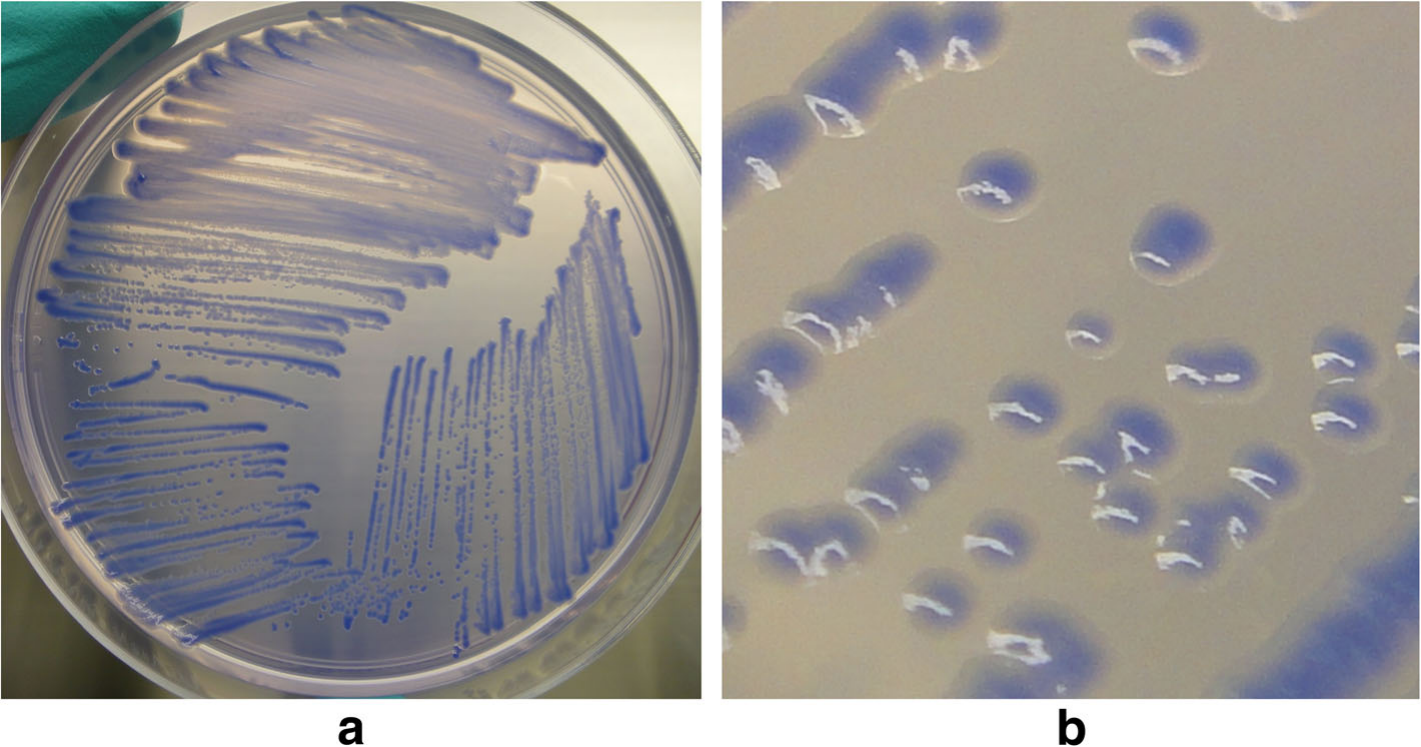
Representative growth of *B. mallei* GTC 3P0003^T^ (**a**) plate view and (**b**) colony view, on BM agar after 72 h incubation at 37 °C

Out of the 38 *B. mallei* strains, 34 strains grew well on BM agar within 48 h, and three strains grew within 72 h. The remaining strain (UCC BURK072) produced colonies after 72 h incubation, but there were fewer colonies on BM agar than the other strains. Most *B. mallei* strains formed circular, purple, smooth, and > 1 mm colonies on BM agar (Fig. 1a and b). All strains of *B. pseudomallei*, *B. thailandensis*, and Bcc also grew on BM agar within 24 h, whereas growths of all 24 non-*Burkholderia* species were completely inhibited during the 72 h incubation period.

### Comparison of selective agars with horse samples

All nasal and skin samples produced numerous commensal bacteria when grown on 5% horse blood agar. Furthermore, these samples produced at least one bacterial colony on Xie’s agar, while 7 and 3 nasal swabs grew bacteria on PC and BM agars, respectively (Table 1). None of the skin swabs grew bacteria on PC or BM agar. Growths of fungi were found from 12 nasal and 7 skin swabs on Xie’s agar, and from 1 nasal and 2 skin swabs on PC agar. No fungi were isolated from any of the tested nasal and skin swabs on BM agar. The following bacterial species were identified on the different agars: Xie’s agar, *Chryseobacterium gleum*, *Bacillus cereus*, *Arthrobacter histidinolovorans*, *Paenibacillus* species, *Bacillus* species, *Enterobacter cloacae*, *Rhizobium radiobacter*, *Staphylococcus saprophyticus*, *Enterococcus casseliflavus*, *Pantoea ananatis*, *Pantoea agglomerans*, *Staphylococcus* species, *Staphylococcus haemolyticus*, *Sphingomonas* species, *Rhizobium* species, and *Escherichia coli*; PC agar, *Klebsiella pneumoniae*, *Pseudomonas putida*, *Pseudomonas koreensis*, and *Pantoea agglomerans*; BM agar, *Herbaspirillum huttiense*.

**Table 1:** Numbers of agars on which at least one colony of bacteria or fungi grew

Medium	Nasal swab (*n* = 34)		Skin swab (*n* = 34)	
	Bacteria	Fungi	Bacteria	Fungi
Xie’s agar	34 (100%)	12 (35.3%)	34 (100%)	7 (20.6%)
PC agar	7 (20.6%)^a^	1 (2.9%)^a^	0 (0%)*	2 (5.9%)
BM agar	3 (8.8%)^a^	0 (0%)^a^	0 (0%)*	0 (0%)*

^a^Significantly lower than number of Xie’s agar

### Comparison of growth efficacy

**Fig. 2:**
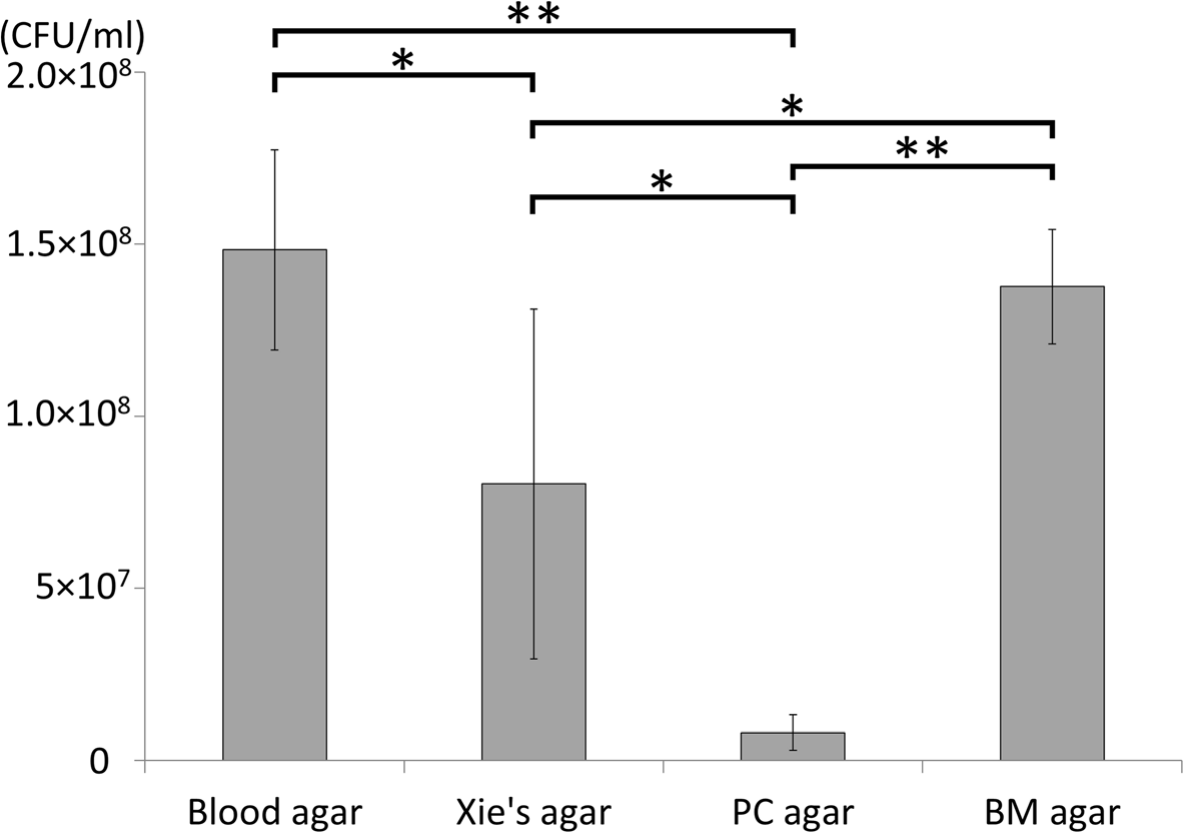
Growth efficiency of *B. mallei* on blood agar and three selective agars. Average colony numbers of McFarland 0.5 standard suspensions are shown. Error bars represent standard deviation (SD). * *p* < 0.01, ** *p* < 0.001

To assess how well BM agar can support *B. mallei* growth we tested three *B. mallei* strains (GTC 3P0003^T^, GTC 3P0016, and GTC 3P0078) for growth on all the three selective agars and blood agar at 37 °C for 72 h. Plating serial dilutions of the same three *B. mallei* cultures on each of the agars, we computed the average bacterial concentration of the original cultures to be 1.48 × 10^8^, 8.03 × 10^7^, 8.03 × 10^6^ and 1.38 × 10^8^ CFU/mL when using blood, Xie’s, PC, and BM agars, respectively (Fig. 2). We noted that the average colony number of three *B. mallei* strains on BM agar was equivalent to that on 5% horse blood agar and was significantly greater than those on the other two agars (*p*-value < 0.05).

### Comparison of culture appearances

**Fig. 3:**
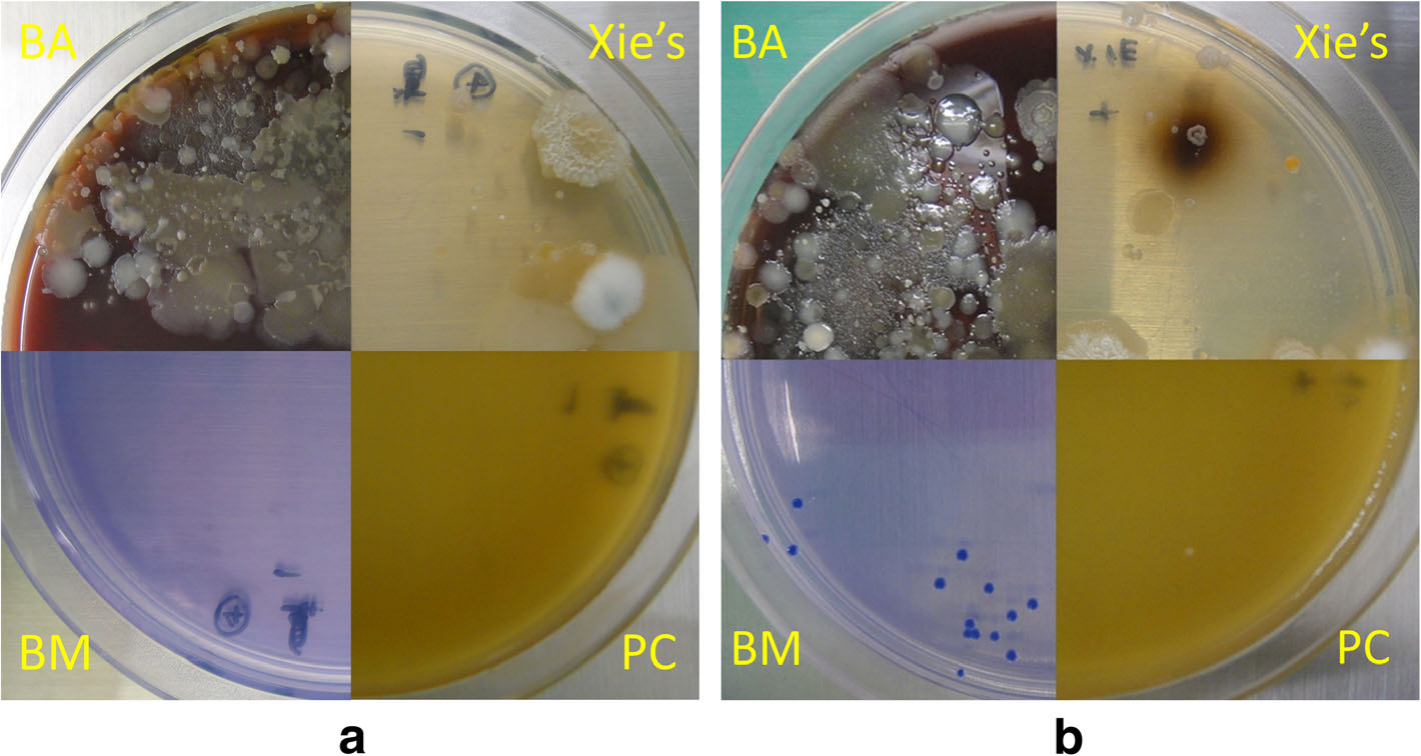
Comparison of culture appearances by using horse nasal swab, (**a**) un-spiked specimen, and (**b**) specimen spiked with *B. mallei* GTC 3P0003^T^. This figure shows that blood agar and Xie’s agar grew other bacteria or fungi, making it difficult to pick up *B. mallei* colonies. BM agar and PC agar can efficiently inhibit growths of non-*B. mallei* isolates. BM agar has the benefit of supporting higher colony numbers, compared with PC agar

Appearances of the four agars by using spiked or non-spiked specimens are shown in Fig. 3. The un-spiked nasal swab did not grow any bacteria or fungi on both PC and BM agars, while several kinds of bacteria or fungi were found on blood agar and Xie’s agar. In the spiked specimen, these commensal bacteria and fungi on Xie’s agar covered *B. mallei* colonies, making it difficult to pick up *B. mallei* colonies. Concerning PC and BM agar, one colony of *B. mallei* was found on PC agar, whereas more than 10 colonies of *B. mallei* were observed on BM agar. The colonies were confirmed as *B. mallei* by the MALDI Biotyper System.

## Discussion

Although glanders has been eradicated from many countries over the last century, several outbreaks of glanders still occurred in the equine population in Asia, Middle-East, Africa, and South America [1, 12]. The international movement of horses increases the risks of spreading exotic diseases, and many cases of disease transfer, including glanders, have been identified [13, 14]. Therefore, quarantines before and after the international transport are quite important. Concerning equine glanders, combination of complement fixation test and western blot is usually performed to check the serological status of horses [3], but a gold standard method for diagnosing this disease remains the isolation of the causative bacterium, *B. mallei* from clinical specimens [2]. One of the important problems of *B. mallei* is its difficulty to isolate the bacteria from environmental samples. Investigators are often unable to isolate *B. mallei* from seropositive or species-specific PCR positive horses [15, 16]. The difficulty of isolating *B. mallei* is mainly attributed to its slow growing character [3]. The bacteria’s growth limitations on commonly used selective media are also considered a reason for the challenges associated with isolating *B. mallei*. For example, *B. pseudomallei*, a genetically closely related species to *B. mallei*, is often isolated on Ashdown’s agar, which uses crystal violet and gentamicin as selective agents [17, 18]. However, *B. mallei* growth generally fails on Ashdown’s agar [9] because the minimum inhibitory concentration of gentamicin for *B. mallei* is significantly lower than that for *B. pseudomallei* [19, 20]. Similarly, MacConkey agar, which is commonly used to isolate Gram-negative and enteric bacilli, is generally not useful for isolating *B. mallei* because the bacteria may or may not grow on this agar [21]. The PC and Xie’s agars are useful in isolating *B. mallei* from contaminated clinical samples [3, 9], but these agars have their own limitations. PC agar could not grow some strains of *B. mallei* and its colonies formed on this agar were very small (< 1 mm) even after 72 h incubation. In addition, the effectiveness of Xie’s agar has not been compared with those of other agars. Therefore, we have developed a new selective agar “BM” to isolate *B. mallei* from equine specimens and compared its selectivity and growth efficiency with the previously recommended selective agars. Although non-selective agars like glycerol dextrose agar and glycerol potato agar can be used to grow *B. mallei*, we excluded these from our study, which focused on using selective agars to isolate *B. mallei* from heavily contaminated samples.

Concerning the formulation of BM agar, glycerol is well known to enhance the growth of *B. mallei* [3], and crystal violet and cycloheximide are used to inhibit some Gram-positive bacteria and fungi. In particular, a combination of antimicrobials is quite important to make a medium with high selectivity. *B. mallei* was reportedly resistant to all the three antimicrobials (ticarcillin, fosfomycin, and polymyxin B) [20, 22]. Ticarcillin has antibacterial activity against Gram-positive bacteria, while polymyxin B acts against Gram-negative bacteria, and fosfomycin affects both Gram-positive and Gram-negative bacteria. Although *B. mallei* was reported to be intrinsically resistant to polymyxin B [22], some *B. mallei* strains showed its relative susceptibility to this antimicrobial in our preliminary test. Specifically, polymyxin B of greater than 70,000 units/L could slow down the growths of *B. mallei* strains (data not shown). Therefore, the concentration of polymyxin B which did not affect the bacterial growths was used in this study. In addition, this view could make an impact on the difference of growth efficacy between BM agar and PC agar as described below, because PC agar contains high concentration of polymyxin B (300,000 units/L).

Our results with bacterial pure cultures showed that the BM agar could allow 34 out of 38 *B. mallei* strains to grow within 48 h. Three of the other four strains needed up to 72 h to be easily picked up from the agar plate. The remaining one grew scantly even after 72 h, indicating that at least 72 h incubation is recommended for isolation of *B. mallei* by using BM agar. Recovery of the slow-growing strain might benefit from pre-enriching the clinical sample or increasing the incubation time. Other *Burkholderia* species tested were also grown on BM agar. Although *Burkholderia* species are not frequently isolated from equine specimens [6, 23, 24], skin samples especially might contain *Burkholderia* species because most species of genus *Burkholderia* naturally exist in soil [25, 26]. Therefore, additional confirmations, such as specific PCR test, are needed after isolating *B. mallei*-suspected colonies from BM agar. Concerning non-*Burkholderia* species, BM agar showed its high specificity by inhibiting the growths of common bacteria frequently found from equine specimens. The results of comparison tests using equine nasal and skin swabs also illustrate the superiority of BM agar. BM agar can inhibit the growth of both bacteria and fungi more efficiently than PC and Xie’s agar. Several Gram-negative bacteria grew on PC agar while both Gram-positive and Gram–negative bacteria grew on Xie’s agar. Although some non-*Burkholderia* species, such as *Herbaspirillum huttiense,* were found on BM agar, its colony number was relatively low and did not complicate the isolation of *B. mallei* from BM agar.

Regarding growth efficiency of *B. mallei*, high effectiveness is also needed for isolating this pathogen because the concentration of *B. mallei* in clinical specimens of the infected equid is low [1, 14]. Our result revealed that BM agar was as effective as the 5% horse blood agar. Comparing to the PC and Xie’s agar, a significantly greater number of *B. mallei* colonies can be grown on BM agar. In addition to BM agar’s specificity, the agar’s high recovery rate makes it useful for isolating *B. mallei* from horse specimens. Therefore, focusing on combined approaches for the detection of *B. mallei* and *B. pseudomallei*, BM agar could be used as a unique selective medium that can efficiently grow these two pathogenic bacteria and inhibit the growth of non-*Burkholderia* species.

## Conclusions

Our study has shown that BM agar is a novel selective agar for effectively isolating *B. mallei* from horse specimens. We believe that this new agar can be used to provide sensitive and selective isolation of *B. mallei* for both diagnosis and future research.

## Methods

### Culture media

BM agar was prepared by combining 5 g of proteose peptone no.3, 10 g of tryptone pancreatic digest of casein, 0.25 g of magnesium sulfate, 1 g of sodium pyruvate, 5 g of sodium chloride, 40 mL of glycerol, 3 mg of crystal violet (3 mL from 0.1% w/v crystal violet in distilled water), 50 mg of cycloheximide (5 mL from 1% w/v cycloheximide in absolute ethanol), 15 g of agar, and distilled water (960 ml). The mixture was sterilized at 121 °C for 15 min. After cooling to 45 °C, the medium was supplemented with three antimicrobials: 16.7 mg of ticarcillin disodium salt (668 μL from 25 mg/ml stock solution), 197.8 mg of fosfomycin sodium (1520 μL from 130 mg/mL stock solution), 40,000 units of polymyxin B (400 μL from 100,000 units/mL stock solution). The formulations of PC agar and Xie’s agar were based on previous reports [9, 11]. Additional file 1 :Table S1 compares the formulations of the BM, PC, and Xie’s agars used in this study.

### Bacteria used in this study

A collection of 68 isolates of *Burkholderia* species and 24 isolates of non-*Burkholderia* species was used for evaluation of BM agar (see Additional file 2 :Table S2). These *Burkholderia* species included 38 *B. mallei*, 5 *B. pseudomallei*, 6 *B. thailandensis*, and 19 strains of *Burkholderia cepacia* complex (Bcc). The non-*Burkholderia* species, which are frequently isolated from horse specimens, were tested in this study and the collection was composed of three strains from each of the following eight bacterial species (total 24 strains): *Pseudomonas aeruginosa*, *Stenotrophomonas maltophilia*, *Escherichia coli*, *Klebsiella pseumoniae*, *Streptococcus zooepidemicus*, *Streptococcus equi*, methicillin-susceptible *Staphylococcus aureus*, and methicillin-resistant *Staphylococcus aureus*.

### Bacterial growth on BM agar with pure cultures

Prior to testing, each of the strains were subcultured onto blood agar or LB agar containing 4% glycerol. For *Burkholderia* species, one colony of each strain was picked up and subcultured onto BM agar. The plates were incubated at 37 °C for 72 h. For non-*Burkholderia* species, each isolate was suspended in 1 mL of saline solution (0.85% w/v) to a turbidity equivalent to a McFarland 0.5 standard (approximately 1.5 × 10^8^ CFU/mL). Each suspension was diluted 1000-fold (approximately 1.5 × 10^5^ CFU/mL) and a 100 μL aliquot of the dilution (approximately 1.5 × 10^4^ CFU/100 μL) was inoculated onto the medium and spread with a plate spreader. Then, the plates were incubated at 37 °C for 72 h. The numbers of bacterial colonies were counted every 24 h.

### Collection and analysis of horse samples

A total of 34 nasal swabs and 34 skin swabs were sampled from 17 healthy horses that were kept by Equine research institute; both sides of the nasal cavity and shoulder skin were sampled from each horse. Each swab was dipped into 500 μL of distilled water, and 100 μL of the suspension was inoculated onto the three selective agars (BM, PC, and Xie’s) and 5% horse blood agar. Growths of bacteria and fungi were checked after incubating at 37 °C for 72 h. Bacterial isolates from horse specimens were identified by matrix assisted laser desorption ionization-time of flight mass spectrometry with MALDI Biotyper System (Bruker Daltonics, Leipzig, Germany), in accordance with the manufacturer’s instructions. The Biotyper software 3.1 and the MALDI Biotyper reference library version 5.0.0.0. were used in this study. The bacterial identifications were interpreted according to the criteria of the manufacturer, namely species level, identification score of ≥2.000; genus level, ≥1.700 to < 2.000; no identification, < 1.700.

### Evaluation of growth efficiencies

Three *B. mallei* strains (GTC 3P0003^T^, GTC 3P0016, and GTC 3P0078) obtained from Gifu Type Culture Collection were used to compare growth efficiencies of *B. mallei* in this study. Each strain was suspended in saline solution (0.85% w/v) to a turbidity equivalent to a McFarland 0.5 standard (original suspension). Ten-fold serial dilutions of the suspensions were inoculated in triplicate onto four agars (5% horse blood agar, BM agar, PC agar, and Xie’s agar) and the average colony counts of the original suspensions were separately calculated for each of the agars after incubation at 37 °C for 72 h.

### Comparison of culture appearances

To compare the appearances of bacteria grown on each of the four agars, *B. mallei* GTC 3P0003^T^ was suspended in 1 mL of phosphate-buffered saline (PBS) to a turbidity equivalent to a McFarland 0.5. Ten-fold serial dilutions of the suspension were prepared. One nasal swab obtained from a healthy horse was dipped into 2.5 mL of PBS. Then, 10 μL of each bacterial dilution and 200 μL of the swab suspension were mixed and 50 μL aliquots of the mixture were inoculated onto the four agars. Appearances of the agars were observed after incubation at 37 °C for 72 h. The same amount of nasal swab suspension was also inoculated onto the four agars without being spiked with *B. mallei*, and bacterial and fungal colonies were checked after incubation for comparison.

### Statistics

A Fisher’s exact test and t-test were conducted by using Excel Statistics ver. 7.0 (Esumi Co., Ltd., Tokyo, Japan) for statistical analysis of comparisons. A *p*-value of < 0.05 was considered to indicate a significant difference.

## Additional files

Additional file 1: **Table S1.** Constituents of three selective agars. (XLSX 12 kb)

Additional file 2: **Table S2.** Bacteria used in this study. (XLSX 14 kb)

## Abbreviations

Bcc: *Burkholderia cepacia* complex
BM agar: *Burkholderia mallei* agar
GTC: Gifu Type Culture Collection
PC agar: *Pseudomonas cepacia* agar
UCC: The United States Army Medical Research Institute for Infectious Diseases’ Unified Culture Collection
